# Supplementary material for: Kinetics and mechanism of selenate and selenite removal in solution by green rust-sulfate
Source: R Soc Open Sci. 2019 Apr 24;6(4):182147. doi: 10.1098/rsos.182147 (PMC6502383; doi:10.1098/rsos.182147)
Supplement: Supplementary Figures [file rsos182147supp1.docx]

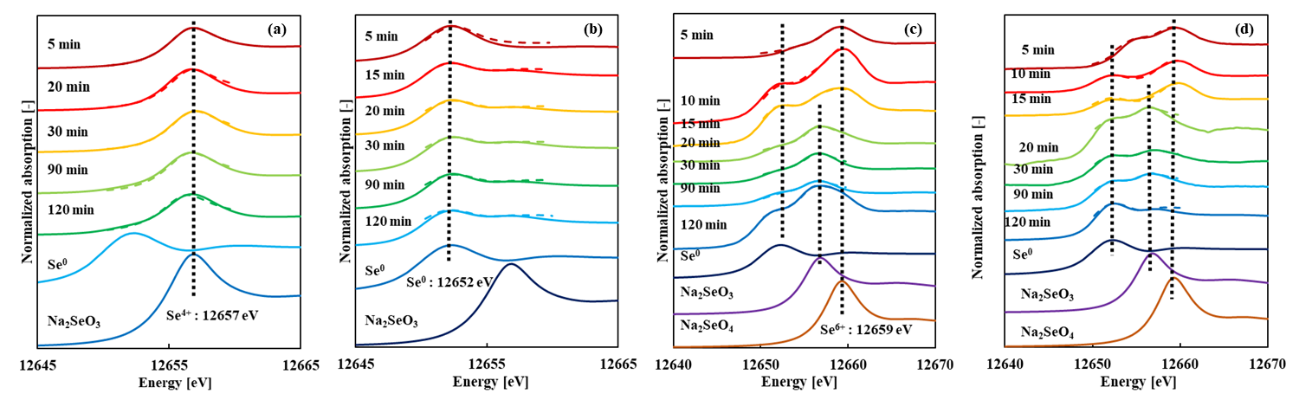


Fig. S1. Normalized Se k-edge XANES spectra of solid samples from removal experiments with Se(IV) pH 8 (a), Se(IV) pH 9 (b), Se(VI) pH 8 (c) and Se(VI) pH 9 (d). The figures show also the XANES patterns of Se(0), Na_2_SeO_3_ and Na_3_SeO_4_ reference materials.


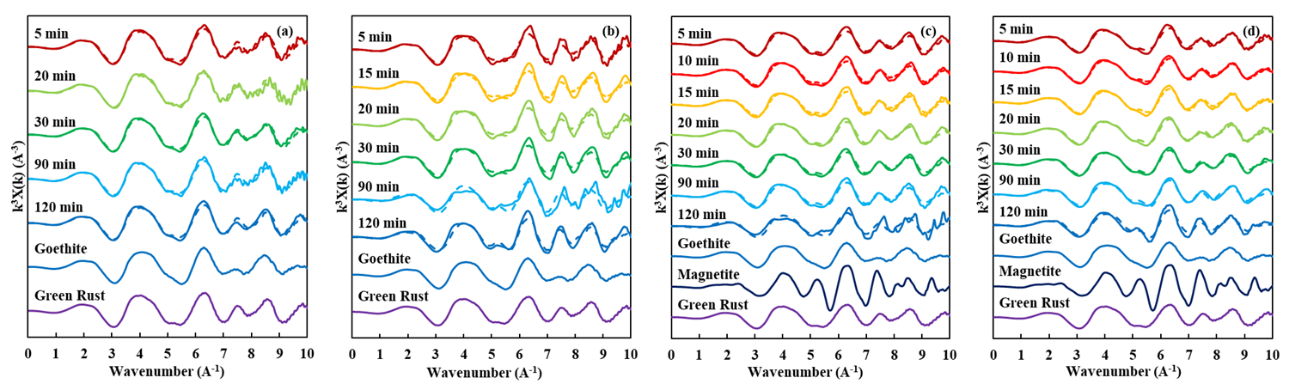


Fig. S2. Fe k-edge k3 weighted EXAFS spectra (b) of solid samples from removal experiments with Se(IV) pH 8 (a), Se(IV) pH 9 (b), Se(VI) pH 8 (c) and Se(VI) pH 9 (d). The figures show also the EXAFS patterns of GR, goethite and magnetite reference materials.
